# Classifying Response Correctness across Different Task Sets: A Machine Learning Approach

**DOI:** 10.1371/journal.pone.0152864

**Published:** 2016-03-31

**Authors:** Thorsten Plewan, Edmund Wascher, Michael Falkenstein, Sven Hoffmann

**Affiliations:** 1 Leibniz Research Centre for Working Environment and Human Factors–Department of Ergonomics, Dortmund, Germany; 2 German Sport University Cologne–Institute of Psychology–Department of Performance Psychology, Cologne, Germany; ghent university, BELGIUM

## Abstract

Erroneous behavior usually elicits a distinct pattern in neural waveforms. In particular, inspection of the concurrent recorded electroencephalograms (EEG) typically reveals a negative potential at fronto-central electrodes shortly following a response error (Ne or ERN) as well as an error-awareness-related positivity (Pe). Seemingly, the brain signal contains information about the occurrence of an error. Assuming a general error evaluation system, the question arises whether this information can be utilized in order to classify behavioral performance within or even across different cognitive tasks. In the present study, a machine learning approach was employed to investigate the outlined issue. Ne as well as Pe were extracted from the single-trial EEG signals of participants conducting a flanker and a mental rotation task and subjected to a machine learning classification scheme (via a support vector machine, SVM). Overall, individual performance in the flanker task was classified more accurately, with accuracy rates of above 85%. Most importantly, it was even feasible to classify responses across both tasks. In particular, an SVM trained on the flanker task could identify erroneous behavior with almost 70% accuracy in the EEG data recorded during the rotation task, and vice versa. Summed up, we replicate that the response-related EEG signal can be used to identify erroneous behavior within a particular task. Going beyond this, it was possible to classify response types across functionally different tasks. Therefore, the outlined methodological approach appears promising with respect to future applications.

## Introduction

In order to adjust behavior rapidly to environmental and one’s own demands it is necessary to carry out action-monitoring permanently. Especially the detection and compensation of erroneous (or undesired) outcomes play an important role in this regard. One neurophysiological correlate of such a response monitoring system can be measured in the electroencephalogram. Shortly following response errors (about 60 ms) a negative potential can be observed at fronto-central electrode positions: the error negativity (Ne [1]) or error-related negativity (ERN [2]). On the neurophysiological level, the anterior cingulate cortex (ACC) seems to be the main structure that is involved in the generation of the Ne, although the supplemental motor area has also been shown to be involved [3–6].

The Ne is being followed by another correlate of such a response monitoring system which is also related to error processing: the error positivity (Pe [1,7]). Typically, the Pe peaks about 200–400 ms following response onset at centro-parietal electrode positions and is assumed to reflect error awareness and evaluation, such that the Pe can be observed, whenever subjects are aware of a recently committed error [7,8]. With respect to the neurophysiological implementation of the Pe, source-localization indicated that the neural regions (e.g. rostral ACC) generating Ne and Pe partly overlap [9]. However, additionally activation of the insula is related to error awareness, indicating an increased awareness with respect to the autonomic reaction to errors [10]. Since the Ne does not vary with error awareness, both components might reflect distinct aspects of error processing [11]. In sum, it is well accepted that both ERP components seem to be closely related to behavioral adaptation.

Indeed, actions are not only adapted via detection and compensation of errors: correct responses are being monitored as well, for example in order to increase motor precision. Accordingly, even after correct responses there is a prominent fronto-central negativity, the correct-related negativity (CRN, see e.g. [12]). Recent evidence pointed to a general response monitoring system (as reflected in Ne and CRN) that is central to the adaptation of actions [13,14]. However, for convenience we use the term Ne throughout the manuscript and specify whether it is referred to correct or incorrect responses. Further evidence for a general response monitoring system can be derived from the finding that CRN and/or Ne both are observable in various types of speeded or simple response tasks [8,13,15–19]. Moreover, seemingly the response modality does not matter, since the Ne is present for example in tasks requiring vocal responses [20].

One theoretical explanation regarding the functional implementation of the Ne is the reinforcement-learning hypothesis (RFL [21]) which is supported by a huge amount of empirical findings (for an overview compare [22]). The RFL proposes that the Ne is triggered whenever an outcome, irrespective of being a response or an event, is worse than expected. Accordingly, the function of the neurophysiologic mechanisms generating the Ne is to initiate remedial action to control errors or undesired outcomes. The RFL is thereby not restricted to errors: it is a general theory about the compensation and adaptation to unexpected outcomes implicating a general functional network linked to the learning and monitoring of stimulus response contingencies [23,24]. However, more recent models and studies provide evidence, that the ACC does not code solely events being worse than expected, but rather the expectancy of events (i.e. if events are expected or not [25]).

Given the accuracy-differentiating character of CRN, Ne, and Pe, the practical question arises, whether this activity (from a statistical point of view) is specific for certain (cognitive) tasks, or can be used as a general classification feature across tasks. If such an adaptive response monitoring system is part of task processing in any task consisting of providing a ‘to-be-evaluated-response’ to some stimuli, classifications across different types of tasks should be feasible. Hence, the first hypothesis of the present study is that the response-related EEG signal can be utilized for the classification of the subject’s current response state, more specifically, whether the current response is correct or not (via classification of Ne and Pe). This idea appears promising as especially the Ne is prominent in the single-trial EEG, and has been shown to be a very reliable ERP, with reliabilities >.85 within subjects [26]. Indeed, a huge number of studies already revealed that it is possible to classify response correctness by utilizing the single-trial Ne (see e.g. [27–29]). In the present study it is aimed to replicate this finding. Going beyond the question whether the Ne can be utilized to classify response correctness, previous research revealed that information about upcoming events can be derived from the individual EEG [30–33]. However, the latter studies were limited to correlates of attentional or perceptual mal-adaptations, not yielding classifications, but rather correlative dependencies. In a recent study a pattern recognition framework has been implemented in order to classify correct and incorrect responses via extraction of EEG features in the time window of Ne and Pe [34] and from these results it has been concluded that the Pe does not contribute much to classification performance. However, in their study the authors did not use the single-trial Ne or Pe, but their algorithm selected automatically the best feature for classification irrespective of electrode position, that is, the whole electrode array was fed into the learning phase and the best predictor was selected. This led to the situation, that sometimes even temporal or lateral electrodes were included, thus it is not clear, whether really the Pe or Ne were selected but rather statistically relevant features [34].

In addition, the core aim of the present study is to test whether the supposed classification of response correctness is stable across tasks. There is much evidence indicating that the mechanisms involved in error processing constitute a general evaluation system [6,12–14,22,23,35,36]. Consequently, it should be possible to use the error-related EEG activity to classify responses accurately across tasks, even if the mechanisms that lead to an error in both tasks are quite different. Indeed, in a previous study it has been shown that the Ne correlates considerably across tasks [19].

To test the assumption that Ne, as well as Pe can be used as general classification features, two different cognitive tasks, namely a mental rotation and a flanker task were conducted and it was investigated whether the response-related activity of one task can be employed to assess behavioral performance in the other. In both experimental tasks actually different types of errors can be assumed; in the flanker task errors are due to lapses of attention [37,38], whereas in the rotation task errors are more likely “mistakes” (i.e. inappropriate target processing). Given that there is a common response, i.e. error monitoring system which is reflected in the Ne, and that error awareness as reflected in the Pe is involved as well, it can be hypothesized that the identification of errors and correct responses should be above chance, despite the fact that in both tasks errors arise due to different cognitive and neural mechanisms [22].

## Methods

The data herein were taken from a previous experimental series [13]. Thus, the standard behavioral and ERP results must not contribute to any meta-analysis. However, we describe the corresponding methods again in order to circumvent cross reading for the reader. Furthermore, the data were re-analyzed, leading to slightly different statistics (but not inferences), which is due to fact that in the previous study the statistics were related to the independent components derived by independent component analysis (ICA) related to the error negativity. In the present study the “pure” ERP was analyzed, since the ICA analysis scheme would go far beyond the scope of the manuscript.

### Participants

The sample consisted of 20 healthy young participants (11 women). Participants were aged between 21 and 27 years (mean = 23.8; SD = 1.9), gave written informed consent prior to participation and received 10, - €/h payment for participation. The local ethics committee of the Leibniz Research Centre for Working Environment and Human Factors approved the study.

### General procedure and experimental design

Participants were seated in an ergonomic seat in front of a 19”-CRT monitor (100 Hz). Responses were given by a button press of the left or right thumb of a force measuring device. The experiment consisted of two tasks each consisting of eight blocks (one training block). Each block consisted of 80 trials. Following each block a break of 20 seconds and after half of the experimental blocks a break of 120 seconds was provided. The initial experiment consisted of a mixed 2 (group) x 2 (task) design with the between subjects factor group (accuracy, speed instruction) and the within subjects factor task (flanker, rotation). The design was fully balanced with respect to group, sequence of tasks, and response side for mirrored/non-mirrored letters. We did not analyze the speed-accuracy manipulation since it was beyond the scope of the present manuscript and, more important, did not lead to any differences with respect to Ne amplitude due to the adaptive deadline (for details compare [13]).

The first task was a modified flanker task [39]. In the center of the screen an arrowhead indicated the button that had to be pressed. This arrowhead was accompanied by two distracting arrowheads below and above which appeared 100 ms prior to target occurrence, which is known to induce maximal distraction [38,40]. These flankers could be congruent (pointing to the same direction) or incongruent (opposite direction). The probability for congruent and incongruent flankers was 50%, respectively.

The second task was a mental rotation task. One out of two letters (F,R) was presented to the participants. This letter was either rotated, mirrored across the main axis or both. Participants had to indicate with a left or right button press of the corresponding thumb if the letter was mirrored or not. The letters were rotated by 0°, 45°,135°, 225° or 315°, resulting in 20 possible stimuli which were presented in random order. Thus, the rotation task was not only much more difficult than the flanker task, it also differed with respect to the degree of stimulus-response mapping.

In both tasks the participants received post-response feedback indicating whether they responded within an appropriate time interval. The feedback consisted of two pictograms: If the participants responded fast enough a yellow pictogram of a smiling face (“smiley”) appeared in the center of the screen. A red angry looking pictogram appeared if they responded too fast or too slow. The deadline for the feedback was adapted block wise. If the error rate in one block (80 trials) was below 8%, the deadline was decreased subtracting one standard deviation from the mean RT of the previous block. In contrast, an error rate above 12% led to an increase of the deadline by adding four standard deviations to the mean RT of the previous block.

### Behavioral data analysis

Error rates of both tasks were compared with each other by means of bootstrap *t*-tests [41]. Observed *t*-values (*t*_obs_), adjusted bootstrap *p*-values (*p*_boot_), and Cohen’s *d* for repeated measures indicating effect sizes are reported [42].

The reaction times (RTs) were analyzed by means of a repeated measures ANOVA with the within subject factors task (flanker, rotation) and response (error, correct), with RTs faster than 100 and slower than 1000 ms being excluded from the analysis. Resulting *F*-values, *p*-values, and partial eta squared are η_p_^2^ reported. Whenever necessary, multiple comparisons were conducted via post-hoc *t*-tests, while the corresponding *p*-values were FDR-adjusted according to the method of Benjamini and Yekutieli [43]. Cohen’s *d* is reported for effect sizes [42].

### EEG data: pre-processing and ERPs

EEG was recorded monopolar (via a QuickAmp, Brain Products, Gilching) from 63-electrodes (FPz, FP1, FP2, AFz, AF7, AF3, AF4, AF8, Fz, F7, F3, F4, F8, FCz, FT7, FC5, FC3, FC1, FC2, FC4, FC6, FT8, T7, C5, C3, Cz, C1, C2, C4, C6, T8, TP7, TP8, CPz, CP5, CP3, CP1, CP2, CP4, CP6, Pz, P7, P3, P1, P2, P4, P8, POz, PO9, PO7, PO3, PO4, PO8, PO10, Oz, O1, O2, M1, M2) with a sampling rate of 500 Hz. The EOG was recorded from the outer canthi and from above and below the right eye (SO2, IO2, LO1, LO2). Data were re-referenced off-line relative to linked mastoids. The EEG was filtered offline using a short non-linear FIR filter (high pass 0.5 Hz, low pass 25 Hz).

Subsequently, the data for each participant were segmented into 1000 ms epochs yielding a temporal data set to which an automated artifact rejection procedure [44] was applied, followed by the ICA AMICA algorithm [45,46]. The automated artifact rejection procedure basically calculates the empirical distribution of all data points across all trials and time points and rejects statistical outliers that are trials consisting of data points exceeding a criterion of 3 standard deviations. The amount of maximal rejected trials was set to 5%. The derived ICA-weights were submitted to the *previous continuous data set*, and independent components representing ocular artifacts were removed by projecting back the mixing matrix with artifact components set to zero [47–49]. Now the pruned data sets were segmented relative to stimulus onset and a second time relative to response onset. Finally, labels coding errors and correct trials were added in order to yield conditional vectors for the machine learning analysis.

For calculation of the ERPs, the data were submitted to the before mentioned automated artifact procedure [44] and the data were averaged for both tasks and response types (errors, correct). The Ne was quantified as a mean voltage (20–100 ms following button press) at FCz, since topographic maps (spherical splines) indicated a maximum at this time point and channel location. The Pe was quantified as mean voltage in the time range 180–250 ms following button press at Cz.

The statistical analysis of task (flanker vs. rotation) and accuracy (correct vs. incorrect) effects on this mean amplitudes consisted of a repeated measures ANOVAs. Due to the 2x2 factorial design the degrees of freedom were 1 for all ANOVAs. Thus, no sphericity correction was applied. We report *F*-values, *p*-values and effects sizes by means of η_p_^2^. If post-hoc *t*-tests were conducted due to significant interactions, we report *t*-values, alpha-adjusted *p*-values [43] and effect sizes by means of Cohens’s *d* for repeated measures [42].

### EEG data: machine learning

The EEG in the present study was analyzed by means of a machine learning approach, i.e. a support vector machine (SVM) was implemented and optimized for classification of single-trial EEG data. SVMs are supervised learning algorithms that aim towards an optimal separation of distinct classes. For that purpose input data is projected into high-dimensional feature space in order to determine a hyperplane which is able separate this data. An SVM trained this way is subsequently able to apply this “knowledge” to new data and hence able to classify it [50,51]. SVMs are used in a wide variety of scientific fields and recently received progressing popularity in classifying brain activation. They were even successfully employed to investigate common neural mechanisms between different tasks. For instance, a shared neural basis for perceptual guesses and free decisions [52] or an involvement of spatial coding in mental arithmetic [53] was investigated this way.

#### Basic analysis scheme

In the analysis EEG data were analyzed separately for both experiments via machine learning in order to classify errors and correct responses. More specifically, there were two analyses: the initial analysis consisted of a classification of errors and correct responses *within* task. The second analysis tested whether it is feasible to identify errors and correct responses *across* tasks.

#### Pattern recognition analysis scheme

Initially, the segmented EEG data was subjected to a feature extraction procedure. According to the hypotheses, feature sets for Ne, Pe, or a combination of both components were constituted for both tasks, resulting in six individual feature sets for each participant. Based on the results of the EEG data five electrodes were selected around the peak position of Ne (i.e. Fz, FCz, FC1, FC2, Cz) and Pe (i.e. Cz, FCz, CPz, C1, C2), respectively. Thus, the Ne and Pe feature set comprised 5 features each. Specifically, the mean voltage on each of the selected electrodes (i.e. average of the EEG signal on that site) was calculated within a time window of 20–100 ms (Ne) and 180–250 ms (Pe). The combined feature set comprised both, Ne and Pe feature set (i.e.10 features). Subsequently, these feature sets are utilized for the classification process by means of a support vector machine (SVM). Therefore, the extracted features of all individual feature sets were linearly scaled in a range between 0 and 1 [54].

In both experimental tasks, errors were less likely to occur than correct responses. Thus, the individual number of errors ranged between 12 and 237 (mean ~104) in the flanker task and 52 and 268 (mean ~128) in the rotation task, respectively. For each participant a corresponding (equal) number of correct response trials was randomly selected for further processing steps. For instance, 100 errors would be matched with 100 (randomly selected) correct responses. Accordingly, the classifier’s accuracy rate is defined as the number of correct classification incidents divided by the total number of classification incidents (i.e. selected trials).

The actual classification procedure was performed using the freely available toolbox *libsvm* [55] as implemented for MATLAB^®^. An SVM with radial basis function (RBF) kernel was employed, since it offers the flexibility to handle linear and nonlinear relations between features and target classes [54,56]. Using a RBF-SVM the penalty parameter C (controlling the cost of misclassifications) and the free parameter of the RBF-kernel γ (determining the shape of the kernel) have to be chosen. Therefore, both parameters were individually determined by successive iteration using a nested grid search approach, i.e. following an initial coarse search a second, finer search is conducted [54]. For *within* task classification the selected features were subjected to a 10-fold cross validation. In other words, the dataset is subdivided into ten parts from which nine parts serve to train the SVM while the remaining part is tested on. This procedure is repeated ten times for each iteration-level of the grid search and results in a mean accuracy rate. More importantly, the selected parameters (C and γ) determine the SVM model that is employed to perform *across* task classifications. For that purpose the SVM was trained on the flanker task data with the previously established parameters and then tested on the rotation task data, and vice versa.

The reliability of cross-validation (*within* task) and *across* task classification was tested using a randomization test procedure [57,58] with 1000 permutations. To ensure that the results are not based on a biased selection of correct trial, the outlined analysis scheme was repeated 10 times using randomly selected samples of correct trials. The accuracy rates associated with the best-found parameters observed in the cross-validation (*within* task) as well as the *across* task classification accuracy rates were used to determine a mean value across these 10 analyses (see above). The resulting mean values are subsequently reported in the results section.

## Results

### Behavioral data

Participants committed significantly fewer errors in the flanker task (13.87%) compared to the rotation task (17.67%, *t*_obs_ = 2.16, *p*_boot_ = .002, *d* = .48). Also, the reaction times were significantly shorter for the flanker task (277 ms, collapsed across correct and erroneous responses) than for the rotation task (441 ms, *F*(1,19) = 77.38, *p* < .001, η_p_^2^ = 0.80). Additionally, subjects responded faster for incorrect (339 ms) than for correct responses (379 ms, *F*(1,19) = 73.68, *p* < .001, η_p_^2^ = 0.80), while there was also a significant interaction of task and response (*F*(1,19) = 43.72, *p* < .001, η_p_^2^ = 0.70).

Multiple comparisons by means of one-sided *t*-tests revealed that in the flanker task responses were significantly faster for incorrect (242 ms) than for correct responses (311 ms, *t*(19) = 22.06, *p* < .001, *d* = 4.93). However, this difference was not significant in the rotation task (436 vs. 446 ms, *t*(19) = 1.15, *p* = .27, *d* = 0.25). Further, incorrect responses in the rotation task took longer compared to incorrect responses in the flanker task (*t*(19) = 8.83, *p* < .001, *d* = .97). This was also true for the correct responses, being slower in the rotation task (*t*(19) = 8.41, *p* < .001, *d* = 1.88).

### EEG data: ERPs

With respect to the ERP (Ne/CRN) at FCz in the time range from 20–100 ms following button press, there was an significant accuracy effect: it was more negative for erroneous compared to correct responses (mean difference = 10.15 μV, *F*(1,19) = 53.42, *p* < .001, η_p_^2^ = 0.74).

Furthermore, there was a significant interaction of task (flanker, rotation) and accuracy (error, correct), *F*(1,19) = 14.69, *p* = .013, η_p_^2^ = 0.44. Accordingly, post-hoc *t*-tests revealed that the difference between correct and erroneous response was more pronounced in the flanker task compared to the rotation task (*t*(19) = 3.83, *p* = .004, *d* = 0.86). Overall, the Ne was more pronounced for the flanker compared to the rotation task (*t*(19) = 2.69, *p* = .012, *d* = 0.60).

**Fig 1:**
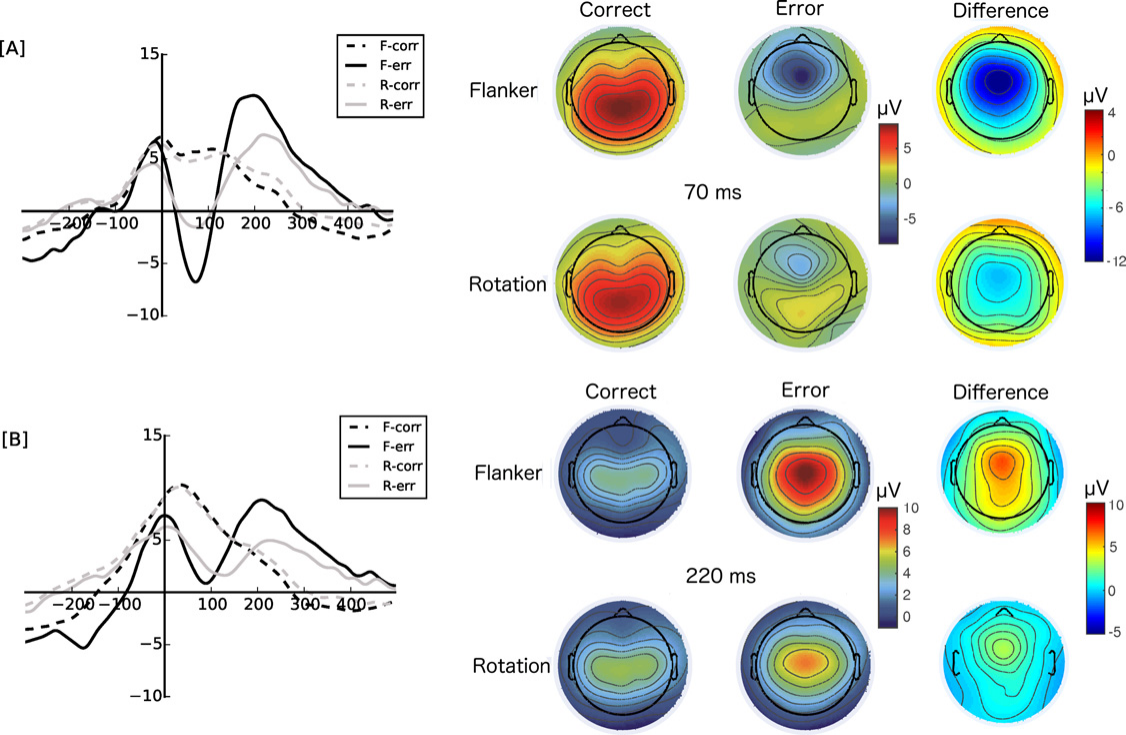
The left panel shows the error negativity at FCz [A] and the error positivity at Cz [B] for errors (-err) and correct (-corr) responses in the flanker task (F-) and rotation task (R-), respectively. The right panel shows the corresponding topographic maps (spherical spline interpolation) for the Ne (70 ms) and Pe peak (220 ms) as well as the difference topography (error-correct) for all conditions (flanker and rotation, correct and errors, respectively).

**Fig 2:**
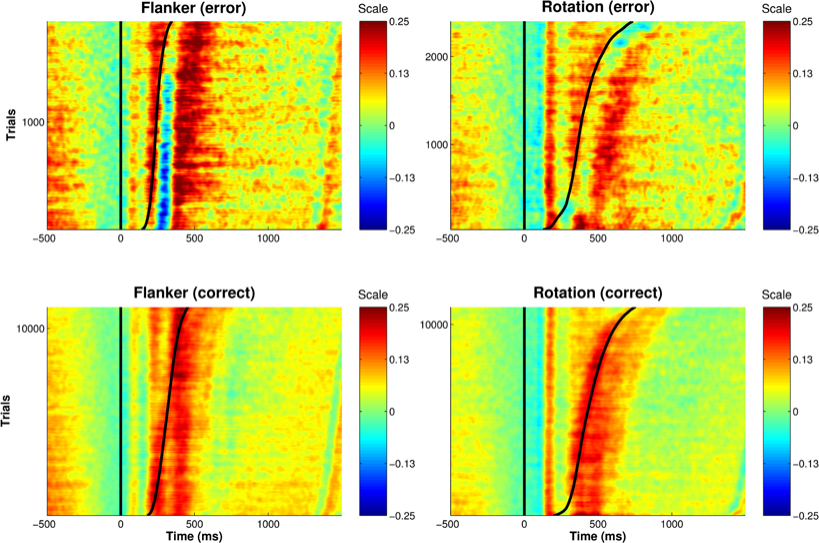
ERP images of the single-trial activity at FCz for trials of all subjects, tasks and accuracy conditions. Note that the single-trial data was normalized and scaled to a range of [-1,1] for each subject prior to concatenating all single-trials. Further, a moving average window (MA) of MA = 40 was applied across trials. The vertical line indicates stimulus onset; the sinusoid line indicates the response. All data were sorted by response time.

With respect to the Pe there was a significant accuracy effect as well (mean difference = 3.97 μV, *F*(1,19) = 8.88, *p* = .007, η_p_^2^ = 0.32). Also, the Pe was more pronounced for the flanker task, compared to the rotation task (mean difference = 1.53 μV, *F*(1,19) = 5.05, *p* = .03, η_p_^2^ = 0.21). However, there was a significant interaction of task and accuracy, *F*(1,19) = 24.12, *p* < .001, η_p_^2^ = 0.56. Post-hoc tests revealed that the difference between errors and correct responses was more pronounced for the flanker task compared to the rotation task (*t*(19) = 3.158, *p* = .007, *d* = 0.71). Overall, the Pe was more pronounced in the flanker task compared to the rotation task (*t*(19) = 4.21, *p* = .001, *d* = 0.94). Figs 1 and 2 summarize the results of the ERP analysis: the ERPs as well as the single-trial signal clearly differ between correct and erroneous responses in both tasks and there is clearly Ne/CRN and Pe activity present in the single-trials.

### EEG data: response classification via machine learning

#### Within task classification

The classification of responses *within* task using an SVM approach was based on 10-fold cross-validation (see methods) and resulted in above chance performance (see Table 1). Trials from the flanker task were correctly classified in about 86% across participants (75–96%, all *p* < 0.05) when both feature sets where employed. Classifier performance slightly decreased when Ne or Pe feature sets were utilized separately. Using only Ne or Pe features resulted in a mean accuracy rate of about 79% (Ne: 61–95%, all *p* < 0.01; Pe: 69–94%, all *p* < 0.05, Tables 2 and 3). A further investigation of the results from the individual subjects indicates however, that there are differences related to the feature sets across participants: Classifiers performance in some participants was clearly driven by Pe features while in other participants the usage of Ne features was beneficial (Table 2 and Table 3).

**Table 1:** Results Flanker Task Cross-Validation.

Subject	Accuracy (mean)	Accuracy (min)	Accuracy (max)	*p* (mean)	*p* (min)	*p* (max)
1	83.088	79.412	86.765	**0**	**0**	**0**
2	88.712	86.364	90.909	**0**	**0**	**0**
3	96.064	94.681	97.872	**0**	**0**	**0**
4	91.966	90.449	93.258	**0**	**0**	**0**
5	80.43	78.146	84.106	**0**	**0**	**0**
6	88.708	87.079	89.888	**0**	**0**	**0**
7	93.152	89.13	95.652	**0**	**0**	**0**
8	95.891	95.05	97.525	**0**	**0**	**0**
9	94	92.593	95.926	**0**	**0**	**0**
10	78.75	70.833	95.833	**0.012**	**0**	**0.031**
11	87.474	85.567	89.691	**0**	**0**	**0**
12	75.485	74.262	78.059	**0**	**0**	**0**
13	82.336	79.918	84.426	**0**	**0**	**0**
14	77.747	75.549	81.868	**0**	**0**	**0**
15	76.039	72.078	81.169	**0**	**0**	**0**
16	73.05	71.631	75.532	**0**	**0**	**0**
17	93.525	91.803	94.672	**0**	**0**	**0**
18	92.059	88.824	95.294	**0**	**0**	**0**
19	76.991	74.537	81.019	**0**	**0**	**0**
20	91.587	87.302	93.651	**0**	**0**	**0**

Bold numbers indicate significance.

Accuracy (mean): indicates the mean accuracy rate derived from 10 analyses

Accuracy (min): indicates the lowest accuracy rate across 10 analyses

Accuracy (max): indicates the highest accuracy rate across 10 analyses

p (mean): mean p-value derived from 10 analyses

p (min): lowest p-value observed across 10 analyses (corresponds to Accuracy (max))

p (max): highest p-value observed across 10 analyses (corresponds to Accuracy (min))

**Table 2:** Results Flanker Task Ne Cross-Validation.

Subject	Accuracy (mean)	Accuracy (min)	Accuracy (max)	*p* (mean)	*p* (min)	*p* (max)
1	75.294	72.059	79.412	**0**	**0**	**0**
2	81.894	78.788	84.091	**0**	**0**	**0**
3	94.894	92.553	97.872	**0**	**0**	**0**
4	88.202	85.955	91.011	**0**	**0**	**0**
5	76.755	74.503	79.801	**0**	**0**	**0**
6	82.64	80.337	84.27	**0**	**0**	**0**
7	92.935	89.13	96.739	**0**	**0**	**0**
8	88.02	86.634	90.099	**0**	**0**	**0**
9	84.741	83.333	85.926	**0**	**0**	**0**
10	80.833	70.833	87.5	**0.002**	**0**	**0.012**
11	81.082	78.351	84.536	**0**	**0**	**0**
12	63.903	61.181	66.456	**0**	**0**	**0**
13	72.049	69.672	75.82	**0**	**0**	**0**
14	60.604	57.967	63.462	**0.001**	**0**	**0.004**
15	67.727	64.935	70.13	**0**	**0**	**0**
16	66.773	64.184	69.149	**0**	**0**	**0**
17	91.721	89.754	93.033	**0**	**0**	**0**
18	85.824	83.529	88.235	**0**	**0**	**0**
19	67.083	62.5	71.296	**0.001**	**0**	**0.001**
20	73.651	69.841	76.984	**0**	**0**	**0**

Bold numbers indicate significance. See Table 1 for further captions.

**Table 3:** Results Flanker Task Pe Cross-Validation.

Subject	Accuracy (mean)	Accuracy (min)	Accuracy (max)	*p* (mean)	*p* (min)	*p* (max)
1	75.196	73.039	76.961	**0**	**0**	**0**
2	73.712	71.97	75.758	**0**	**0**	**0**
3	78.83	75.532	82.979	**0**	**0**	**0**
4	83.652	80.899	85.955	**0**	**0**	**0**
5	73.841	71.854	75.828	**0**	**0**	**0**
6	84.382	83.146	85.955	**0**	**0**	**0**
7	71.957	67.391	75	**0.001**	**0**	**0.004**
8	93.812	92.079	95.05	**0**	**0**	**0**
9	86.815	85.556	88.519	**0**	**0**	**0**
10	76.25	66.667	83.333	**0.013**	**0.001**	0.058
11	81.907	78.866	84.536	**0**	**0**	**0**
12	74.473	73.84	75.738	**0**	**0**	**0**
13	76.639	73.77	79.918	**0**	**0**	**0**
14	78.462	75	81.593	**0**	**0**	**0**
15	74.61	71.429	77.273	**0**	**0**	**0**
16	69.22	66.667	72.34	**0**	**0**	**0**
17	84.385	81.967	86.066	**0**	**0**	**0**
18	87.294	84.118	88.824	**0**	**0**	**0**
19	74.306	71.296	77.315	**0**	**0**	**0**
20	90.952	88.889	92.857	**0**	**0**	**0**

Bold numbers indicate significance. See Table 1 for further captions.

A similar pattern of results was observed for rotation task classification, even though the overall accuracy was slightly worse 75% (58–85%, all *p* < .01, Table 4). Again, classifier performance was diminished using Ne features (69%: 54–80%, all *p* < .01; except of one participant *p* = .074, Table 5) and Pe features (71%: 58–85%, all *p* < .05, Table 6) separately. As observed for the flanker task classification, in some individuals the classifier was more accurate using Ne features, while in others Pe features led to better results. Thus, data derived from both tasks indicate, that there is no advantage for either feature set on its own but the combination leads to a general improvement.

**Table 4:** Results Rotation Task Cross-Validation.

Subject	Accuracy (mean)	Accuracy (min)	Accuracy (max)	*p* (mean)	*p* (min)	*p* (max)
1	74	70.8	76.4	**0**	**0**	**0**
2	81.429	77.273	85.065	**0**	**0**	**0**
3	83.162	80.882	86.765	**0**	**0**	**0**
4	71.681	67.672	76.293	**0**	**0**	**0**
5	74.029	71.471	76.176	**0**	**0**	**0**
6	84.545	81.061	87.121	**0**	**0**	**0**
7	83.654	78.846	87.5	**0**	**0**	**0**
8	76.176	73.529	78.992	**0**	**0**	**0**
9	85.13	83.043	87.826	**0**	**0**	**0**
10	72.05	67.5	74	**0**	**0**	**0**
11	67.047	64.961	68.504	**0**	**0**	**0**
12	60.97	59.328	62.5	**0**	**0**	**0**
13	66.667	63.333	70.303	**0**	**0**	**0**
14	72.637	68.681	75.549	**0**	**0**	**0**
15	81.833	78.889	84.444	**0**	**0**	**0**
16	78.333	74.667	81.333	**0**	**0**	**0**
17	67.462	64.583	69.508	**0**	**0**	**0**
18	84.747	81.818	88.384	**0**	**0**	**0**
19	57.62	55.529	58.654	**0.008**	**0**	**0.039**
20	83.462	80.128	88.462	**0**	**0**	**0**

Bold numbers indicate significance. See Table 1 for further captions.

**Table 5:** Results Flanker Task Ne Cross-Validation.

Subject	Accuracy (mean)	Accuracy (min)	Accuracy (max)	*p* (mean)	*p* (min)	*p* (max)
1	66.16	62.8	68.4	**0**	**0**	**0**
2	76.948	74.026	79.87	**0**	**0**	**0**
3	79.559	76.471	82.353	**0**	**0**	**0**
4	68.707	65.517	74.569	**0**	**0**	**0**
5	66.853	63.529	68.824	**0**	**0**	**0**
6	73.864	65.909	78.788	**0**	**0**	**0**
7	75	69.231	79.808	**0**	**0**	**0**
8	69.664	66.807	72.689	**0**	**0**	**0**
9	73.609	70.87	78.696	**0**	**0**	**0**
10	71.95	66.5	76	**0**	**0**	**0**
11	66.89	63.78	70.866	**0**	**0**	**0**
12	54.086	52.612	56.716	0.074	**0.004**	0.152
13	63.515	60.909	66.061	**0.001**	**0**	**0.001**
14	63.819	61.538	66.209	**0.001**	**0**	**0.001**
15	78.389	75	81.111	**0**	**0**	**0**
16	68.933	65.333	76.667	**0.001**	**0**	**0.002**
17	62.67	61.174	64.015	**0**	**0**	**0**
18	72.727	69.192	75.253	**0**	**0**	**0**
19	58.558	55.048	61.058	**0.008**	**0**	**0.031**
20	65.641	62.821	69.231	**0.001**	**0**	**0.002**

Bold numbers indicate significance. See Table 1 for further captions.

**Table 6:** Results Rotation Task Pe Cross-Validation.

Subject	Accuracy (mean)	Accuracy (min)	Accuracy (max)	*p* (mean)	*p* (min)	*p* (max)
1	71.36	68	73.6	**0**	**0**	**0**
2	69.156	64.935	74.026	**0.001**	**0**	**0.001**
3	79.779	75.735	85.294	**0**	**0**	**0**
4	69.181	67.241	70.259	**0**	**0**	**0**
5	71	68.529	72.941	**0**	**0**	**0**
6	78.333	75	83.333	**0**	**0**	**0**
7	78.269	75.962	81.731	**0**	**0**	**0**
8	73.908	68.908	76.05	**0**	**0**	**0**
9	79.391	76.087	82.609	**0**	**0**	**0**
10	61.85	56.5	65.5	**0.009**	**0**	**0.07**
11	60.079	54.331	64.567	**0.019**	**0**	0.11
12	61.045	59.515	63.06	**0**	**0**	**0**
13	64.818	62.121	70	**0**	**0**	**0**
14	71.071	68.956	73.352	**0**	**0**	**0**
15	76.611	71.667	80.556	**0**	**0**	**0**
16	73.6	71.333	80	**0**	**0**	**0**
17	59.451	57.386	61.553	**0.001**	**0**	**0.004**
18	84.747	82.828	88.384	**0**	**0**	**0**
19	58.317	55.769	61.538	**0.006**	**0**	**0.027**
20	84.487	78.846	87.821	**0**	**0**	**0**

Bold numbers indicate significance. See Table 1 for further captions.

#### Across task classification

In case that there are generalizable neural patterns between flanker and rotation task a classification across tasks should be feasible. Indeed, the SVM classifier was still able to classify responses above chance level *across* tasks in most cases. An SVM trained on flanker task data correctly classified responses from the rotation task in about 67% (55–80%, Table 7). The permutation test indicated significance for ten participants (*p* < .05) and strong trends in additional seven participants (*p* < .11), while less reliable accuracy was only observed for 3 participants (i.e., *p* = .12; *p* = .12; *p* = .14). In cases the SVM was trained with Ne features only, the classifier’s performance drops to about 61% (50–74%, see *p*-values in Table 8) and training with Pe features only slightly improves results to 64% (49–82%, see *p*-values in Table 9).

**Table 7:** Results Across Task: Train Flanker, Classify Rotation.

Subject	Accuracy (mean)	Accuracy (min)	Accuracy (max)	*p* (mean)	*p* (min)	*p* (max)	trials correct (mean)	trials correct (min)	trials correct (max)	trials (total)
1	68	65.6	71.6	**0.006**	**0**	**0.032**	170	164	179	250
2	74.416	71.429	77.273	**0.015**	**0**	0.052	114.6	110	119	154
3	79.779	72.059	83.088	**0.007**	**0**	**0.030**	108.5	98	113	136
4	62.069	59.914	65.086	0.081	**0.043**	0.125	144	139	151	232
5	66.441	59.706	69.706	**0.005**	**0**	**0.014**	225.9	203	237	340
6	79.015	72.727	83.333	**0.006**	**0**	**0.019**	104.3	96	110	132
7	70.673	63.462	75.962	0.072	**0.004**	0.321	73.5	66	79	104
8	68.361	63.866	71.429	**0.043**	**0**	0.208	162.7	152	170	238
9	75.783	73.478	77.826	**0.012**	**0**	0.056	174.3	169	179	230
10	61.65	54.5	66	0.120	**0.039**	0.372	123.3	109	132	200
11	60.63	58.661	63.386	0.078	**0.002**	0.327	154	149	161	254
12	57.593	56.716	58.396	0.080	**0.005**	0.144	308.7	304	313	536
13	58.303	56.667	61.212	0.069	**0.004**	0.129	192.4	187	202	330
14	61.593	57.967	65.11	0.082	**0.018**	0.224	224.2	211	237	364
15	67.333	56.667	73.333	**0.038**	**0**	0.126	121.2	102	132	180
16	60.533	53.333	68	0.105	**0.015**	0.331	90.8	80	102	150
17	56.307	54.356	59.091	0.115	**0.039**	0.217	297.3	287	312	528
18	77.374	74.242	81.313	**0.017**	**0**	0.056	153.2	147	161	198
19	54.543	51.923	56.01	0.144	**0.043**	0.259	226.9	216	233	416
20	77.436	73.077	82.051	**0.013**	**0**	0.073	120.8	114	128	156

Bold numbers indicate significance.

Trials correct (mean): mean number of correctly classified trials across 10 analyses

Trails correct (min): lowest number of correctly classified trials in 10 analyses

Trials correct (max): highest number of correctly classified trials in 10 analyses

Trials (total): Number of instances (i.e. error trials + (equal) number of correct trial)

See Table 1 for further captions.

**Table 8:** Results Across Task Ne: Train Flanker, Classify Rotation.

Subject	Accuracy (mean)	Accuracy (min)	Accuracy (max)	*p* (mean)	*p* (min)	*p* (max)	trials correct (mean)	trials correct (min)	trials correct (max)	trials (total)
1	61.92	58.8	65.6	**0.029**	**0.001**	0.125	154.8	147	164	250
2	72.208	68.182	75.325	**0.018**	**0.003**	**0.044**	111.2	105	116	154
3	73.603	59.559	80.147	**0.023**	**0**	0.200	100.1	81	109	136
4	59.914	56.897	63.793	0.230	0.16	0.398	139	132	148	232
5	63.824	61.176	65	**0.020**	**0.001**	**0.047**	217	208	221	340
6	65.606	59.091	74.242	0.072	**0.004**	0.223	86.6	78	98	132
7	64.038	60.577	67.308	0.138	**0.002**	0.25	66.6	63	70	104
8	69.328	65.546	72.689	**0.002**	**0**	**0.007**	165	156	173	238
9	64.043	61.304	65.652	0.088	**0.012**	0.206	147.3	141	151	230
10	63	50	70	0.162	**0.013**	0.498	126	100	140	200
11	61.535	56.299	65.748	0.070	**0**	0.186	156.3	143	167	254
12	52.817	51.493	54.104	0.071	**0.012**	0.147	283.1	276	290	536
13	57.242	54.848	60.303	**0.050**	**0.002**	0.129	188.9	181	199	330
14	49.423	45.604	53.846	0.501	0.165	0.786	179.9	166	196	364
15	64.778	56.667	70	0.074	**0.015**	0.222	116.6	102	126	180
16	55.6	48	62	0.263	**0.022**	0.618	83.4	72	93	150
17	53.277	51.894	54.735	0.271	0.195	0.39	281.3	274	289	528
18	64.293	62.121	66.667	0.168	**0.025**	0.292	127.3	123	132	198
19	51.827	48.798	54.567	0.268	**0.032**	0.615	215.6	203	227	416
20	60.128	53.846	66.667	0.092	**0**	0.24	93.8	84	104	156

Bold numbers indicate significance. See Tables 1 and 7 for further captions.

**Table 9:** Results Across Task Pe: Train Flanker, Classify Rotation.

Subject	Accuracy (mean)	Accuracy (min)	Accuracy (max)	*p* (mean)	*p* (min)	*p* (max)	trials correct (mean)	trials correct (min)	trials correct (max)	trials (total)
1	65.32	63.6	67.2	0.056	**0.012**	0.109	163.3	159	168	250
2	61.234	55.195	66.234	0.101	**0.007**	0.247	94.3	85	102	154
3	69.485	62.5	77.941	0.064	**0.003**	0.216	94.5	85	106	136
4	61.466	58.621	66.379	0.064	**0.004**	0.149	142.6	136	154	232
5	66.706	64.412	69.412	**0.018**	**0.003**	**0.044**	226.8	219	236	340
6	73.485	67.424	76.515	**0.048**	**0.004**	0.116	97	89	101	132
7	62.019	56.731	67.308	0.194	**0.042**	0.511	64.5	59	70	104
8	67.815	65.546	71.008	**0.013**	**0**	**0.044**	161.4	156	169	238
9	73.261	69.565	75.217	**0.034**	**0**	0.106	168.5	160	173	230
10	52.9	44.5	57.5	0.329	**0.05**	0.806	105.8	89	115	200
11	52.48	48.031	55.118	0.246	**0.016**	0.692	133.3	122	140	254
12	57.239	55.037	60.448	0.145	**0.025**	0.286	306.8	295	324	536
13	56.273	53.333	57.576	0.181	**0.039**	0.379	185.7	176	190	330
14	65.055	61.538	67.308	**0.039**	**0**	0.093	236.8	224	245	364
15	65.667	60	75	0.088	**0.001**	0.207	118.2	108	135	180
16	63.6	59.333	67.333	**0.041**	**0.002**	0.085	95.4	89	101	150
17	48.712	47.159	50.758	0.613	0.384	0.818	257.2	249	268	528
18	82.475	80.303	84.848	**0.001**	**0**	**0.003**	163.3	159	168	198
19	55.072	52.885	56.731	0.159	**0.046**	0.388	229.1	220	236	416
20	77.821	67.308	85.897	**0.029**	**0**	0.230	121.4	105	134	156

Bold numbers indicate significance. See Tables 1 and 7 for further captions.

Reversing the train-test order of the SVM (train on rotation task data, test flanker task data) resulted in a mean accuracy rate of about 75% (58–88%, Table 10). Performance was significant for 13 participants (*p* < .05), with a trend towards significance in 6 participants (*p* < .11), while responses were not reliably classified in a remaining participant (*p* = .14). Training the classifier on Ne and Pe features only yielded similar accuracy rates (Ne: 68% (50–87%); Pe: 68% (58–90%), see *p*-values in Tables 11 and 12). Again, in both types of analysis it became evident that Ne and Pe do not have the same predictability within individual participants.

**Table 10:** Results Across Task: Train Rotation, Classify Flanker.

Subject	Accuracy (mean)	Accuracy (min)	Accuracy (max)	*p* (mean)	*p* (min)	*p* (max)	trials correct (mean)	trials correct (min)	trials correct (max)	trials (total)
1	70.294	67.647	72.549	0.078	**0.01**	0.211	143.4	138	148	204
2	79.545	74.242	85.606	**0.006**	**0**	**0.036**	105	98	113	132
3	84.255	73.404	94.681	**0.026**	**0**	0.114	79.2	69	89	94
4	67.247	50.562	76.404	0.106	**0**	0.467	119.7	90	136	178
5	73.874	66.556	76.49	**0.022**	**0.001**	0.090	223.1	201	231	302
6	68.596	50.562	78.09	0.100	**0**	0.487	122.1	90	139	178
7	86.196	80.435	92.391	**0.017**	**0**	0.072	79.3	74	85	92
8	86.782	76.733	91.584	**0.018**	**0.001**	0.081	175.3	155	185	202
9	88.444	82.222	92.593	**0.001**	**0**	**0.006**	238.8	222	250	270
10	74.583	62.5	83.333	0.051	**0**	0.248	17.9	15	20	24
11	78.969	74.227	84.536	**0.021**	**0**	0.069	153.2	144	164	194
12	67.975	60.97	72.574	**0.024**	**0**	0.090	322.2	289	344	474
13	69.057	50	77.869	0.087	**0.005**	0.549	168.5	122	190	244
14	69.835	67.582	73.352	**0.007**	**0**	**0.026**	254.2	246	267	364
15	68.182	61.688	72.727	**0.030**	**0**	0.072	105	95	112	154
16	58.05	52.128	60.638	0.102	**0.047**	0.312	163.7	147	171	282
17	72.459	60.246	79.918	0.137	**0.005**	0.303	176.8	147	195	244
18	83.412	79.412	88.235	**0.020**	**0**	0.079	141.8	135	150	170
19	69.167	61.574	75.926	**0.043**	**0**	0.160	149.4	133	164	216
20	86.825	83.333	91.27	**0.018**	**0**	0.133	109.4	105	115	126

Bold numbers indicate significance. See Tables 1 and 7 for further captions.

**Table 11:** Results Across Task Ne: Train Rotation, Classify Flanker.

Subject	Accuracy (mean)	Accuracy (min)	Accuracy (max)	*p* (mean)	*p* (min)	*p* (max)	trials correct (mean)	trials correct (min)	trials correct (max)	trials (total)
1	60.686	55.392	67.647	0.195	**0.07**	0.398	123.8	113	138	204
2	73.939	70.455	78.03	**0.043**	**0.002**	0.171	97.6	93	103	132
3	86.809	81.915	93.617	0.067	**0**	0.220	81.6	77	88	94
4	70	53.371	75.843	0.169	**0.006**	0.366	124.6	95	135	178
5	73.046	68.874	76.821	**0.036**	**0**	0.153	220.6	208	232	302
6	65.281	53.371	78.652	0.162	**0.005**	0.425	116.2	95	140	178
7	80	71.739	90.217	0.139	**0.028**	0.285	73.6	66	83	92
8	83.267	80.198	87.624	**0.049**	**0**	0.210	168.2	162	177	202
9	76.593	73.704	80.37	**0.041**	**0**	0.133	206.8	199	217	270
10	73.333	62.5	83.333	0.063	**0**	0.174	17.6	15	20	24
11	75.773	73.196	79.897	**0.048**	**0**	0.255	147	142	155	194
12	59.093	51.477	62.236	0.123	**0.004**	0.303	280.1	244	295	474
13	62.705	58.607	68.033	0.058	**0.011**	0.134	153	143	166	244
14	50.824	45.879	56.044	0.461	0.115	0.864	185	167	204	364
15	64.351	57.792	68.831	**0.022**	**0**	0.087	99.1	89	106	154
16	49.645	43.617	55.319	0.513	0.096	0.947	140	123	156	282
17	76.025	69.672	80.738	0.112	**0.017**	0.302	185.5	170	197	244
18	73.412	67.647	77.647	0.182	0.098	0.298	124.8	115	132	170
19	49.861	45.37	58.333	0.515	0.084	0.890	107.7	98	126	216
20	63.492	52.381	69.048	0.103	**0.014**	0.475	80	66	87	126

Bold numbers indicate significance. See Tables 1 and 7 for further captions.

**Table 12:** Results Across Task Pe: Train Rotation, Classify Flanker.

Subject	Accuracy (mean)	Accuracy (min)	Accuracy (max)	*p* (mean)	*p* (min)	*p* (max)	trials correct (mean)	trials correct (min)	trials correct (max)	trials (total)
1	67.304	64.216	71.569	0.086	**0**	0.265	137.3	131	146	204
2	65.909	55.303	71.212	**0.042**	**0.002**	0.158	87	73	94	132
3	71.809	67.021	79.787	**0.009**	**0.001**	**0.039**	67.5	63	75	94
4	61.966	55.056	68.539	0.156	**0.028**	0.291	110.3	98	122	178
5	66.821	64.57	68.543	0.084	**0.025**	0.199	201.8	195	207	302
6	56.966	50.562	67.416	0.338	**0.08**	0.514	101.4	90	120	178
7	66.087	57.609	71.739	0.072	**0.003**	0.227	60.8	53	66	92
8	76.98	68.812	84.653	0.115	**0.032**	0.184	155.5	139	171	202
9	81.926	77.037	85.926	**0.020**	**0**	**0.046**	221.2	208	232	270
10	57.917	37.5	75	0.340	**0.017**	0.749	13.9	9	18	24
11	59.639	45.876	65.979	0.193	**0.001**	0.77	115.7	89	128	194
12	66.118	61.603	71.308	**0.031**	**0.001**	0.091	313.4	292	338	474
13	66.352	56.148	72.131	0.102	**0.002**	0.334	161.9	137	176	244
14	74.231	71.429	76.374	**0.006**	**0**	**0.026**	270.2	260	278	364
15	67.013	64.935	70.13	0.055	**0.003**	0.175	103.2	100	108	154
16	58.085	54.255	62.766	0.109	**0.01**	0.251	163.8	153	177	282
17	58.484	50.41	62.705	0.360	0.194	0.566	142.7	123	153	244
18	82.235	78.235	87.059	**0.005**	**0**	**0.015**	139.8	133	148	170
19	64.907	60.185	74.074	0.086	**0.013**	0.225	140.2	130	160	216
20	89.603	87.302	92.063	**0.004**	**0**	**0.022**	112.9	110	116	126

Bold numbers indicate significance. See Tables 1 and 7 for further captions.

## Discussion

The present study addressed the question whether it is possible to classify response correctness (i.e. separating correct from erroneous responses) within and across two different cognitive tasks using a machine learning approach. For this purpose, data were re-analyzed from another study [13]. In this study, participants conducted a flanker and a mental rotation task. The re-analysis revealed the same data pattern with respect to the behavioral data like in the previous publication [13].

This behavioral result pattern was accompanied by a significant Pe and Ne component in erroneous trials: both were clearly discernable, even though the difference between correct and incorrect trials was much more elaborated within the flanker task condition. Thus, both components (Ne and Pe) subsequently served as features for the SVM. Overall, the SVM yielded high accuracy rates up to over 80%. As expected, responses were identified most accurately *within* particular task sets, whereby responses could be classified with higher precision in the flanker task. More importantly, it was possible to train an SVM with data from one task and to classify responses *across* tasks. In both cases (*within* and *across* tasks) accuracy classification was most precise using a combination of Ne and Pe features.

It has previously been demonstrated that EEG signal can be utilized to classify response correctness utilizing single-trial Ne and Pe (e.g. [27–29] and also to derive predictions about upcoming behavior and errors [30,32,33,52]. Thus, the present study replicates some findings in this regard. However, there is information besides the Ne that can enhance the separation of correct from erroneous responses. Indeed, it has recently been shown that the signal as reflected in the Ne is constituted by at least two processes: a centrally distributed error-sensitive factor and an outcome-independent factor contributing to both Ne and CRN [59]. Furthermore, the neural mechanisms involved in generating the Pe seem to differentiate between perceived and unperceived errors [60]. In this regard, this is the first time it is shown that the Pe appears to be stable *across* tasks as well (despite the fact that the degree of error awareness differs in both tasks). Thus, though being modulated by task features, the Pe can be utilized to classify effects of error awareness *across* tasks. In sum, the present results strengthen the notion, that both, Ne and Pe, provide error related information, usable for precise classifications.

Furthermore, the SVM trained on the rotation task and tested on the flanker tasks was more accurate compared to the opposite case (i.e. the SVM trained on the flanker task and tested on the rotation task). This adds evidence to the assumption that the rotation task per se is more difficult, most likely conveyed by attenuated response monitoring [4,61]. However, as responses in the flanker task condition are classified with higher accuracy, the supposed error-monitoring process might as well reflect the classifiers’ capability to better distinguish correct from erroneous responses in the “easier” flanker task.

With respect to the assumption of Ne and Pe reflecting a general response evaluation system, a recent investigation on convergent validity of error-related brain activity [19] revealed that there was overlapping variation in error-related brain activity across three different tasks (i.e. flanker, stroop, Go/No-Go task). However, in their study Riesel and colleagues used correlational measures whereas in the present investigation a classification approach was utilized. This offers the advantage, that if the SVM is being trained on one task, the derived classifier can be used in *another* task to identify correct erroneous and correct responses. Apparently, Ne and Pe constitute informative features with respect to response behavior *within* a particular task setting but also *across* tasks that might be utilized as a general classifier. The latter might have important implications, for example it could be tested whether the Ne as well as Pe can be employed with respect to clinical applications [31].

Also, the results of the present study partly challenge the finding of Ventouras and colleagues [34], who concluded from their finding that the Pe does not improve classification performance. This competing result might be due to the different classification scheme: the Pe as well as Ne were selected a priori and fed into the learning phase, whereas in the work of Ventouras and colleagues [34] the most relevant statistical feature was selected, irrespective of electrode position. Thus, it is questionable, whether actually the Pe was selected for classification. Furthermore, in their study a flanker task was utilized. Typically, in this kind of task participants have a clear impression of committing an error. Since the Pe has often been related to error awareness [7,8], this notion is unexpected and not in line with the present results. In contrast, we found a discernable Pe in single trials (compare Fig 2) that improved classification accuracy.

However, the suggested approach has also some limitation. The performance of the classification is likely due to the fact that both experiments were conducted in a successive order, and thus the EEG signal is relatively stable. It is well known that the EEG signal is quite sensitive to noise, biorhythms, arousal and many other factors. Thus, the features derived for instance on one day might be not as predictive if the second task was conducted on another day, even if exact the same experimental setup was used. Thus, it has yet to be tested, whether these signals can even be utilized not only to classify across tasks, but also to predict behavior if the tasks are not conducted immediately following each other.

In sum, the present findings replicate the role of Ne and Pe as reflections of basic error monitoring processes. What is more, those monitoring processes obviously apply *across* different cognitive tasks, even though a reliable classification was not observed in all participants. However, what goes beyond classification is that the features of one task are predictive with respect to the performance in a following task. Thus, the error related EEG signal might be used to predict behavior in interleaved, i.e. subsequent conducted tasks. Accordingly, the outlined approach is also promising with respect towards an application oriented perspective, for instance within a brain-computer interface framework. Based on the present results it appears at least possible that upcoming errors might be detected even before they were committed, if tasks are conducted subsequently. Finally, the applied machine learning approach was successful in demonstrating that a functional mechanism of one task can be identified in another task. Moreover, the combination of advanced data mining strategies and EEG analysis provides the opportunity to test whether different cognitive tasks depend on similar neural mechanisms.
